# Uncovering the Activity of Alkaline Earth Metal Hydrogenation Catalysis Through Molecular Volcano Plots

**DOI:** 10.1007/s11244-021-01480-7

**Published:** 2021-08-25

**Authors:** Shubhajit Das, Bart De Tobel, Mercedes Alonso, Clémence Corminboeuf

**Affiliations:** 1 Laboratory for Computational Molecular Design, Institute of Chemical Sciences and Engineering, Ecole Polytechnique Fedéralé de Lausanne (EPFL), Lausanne, 1015 Switzerland; 2 Eenheid Algemene Chemie (ALGC), Vrije Universiteit Brussel (VUB), Pleinlaan 2, Brussels, 1050 Belgium

**Keywords:** Alkaline earth metal catalysis, Hydrogenation, Molecular volcano plots, Linear scaling relationships, DFT

## Abstract

Recent advances in alkaline earth (Ae) metal hydrogenation catalysis have broadened the spectrum of potential catalysts to include candidates from the main group, providing a sustainable alternative to the commonly used transition metals. Although Ae-amides have already been demonstrated to catalyze hydrogenation of imines and alkenes, a lucid understanding of how different metal/ligand combinations influence the catalytic activity is yet to be established. In this article, we use linear scaling relationships and molecular volcano plots to assess the potential of the Ae metal-based catalysts for the hydrogenation of alkenes. By analyzing combinations of eight metals (mono-, bi-, tri-, and tetravalent) and seven ligands, we delineate the impact of metal-ligand interplay on the hydrogenation activity. Our findings highlight that the catalytic activity is majorly determined by the charge and the size of the metal ions. While bivalent Ae metal cations delicately regulate the binding and the release of the reactants and the products, respectively, providing the right balance for this reaction, ligands play only a minor role in determining their catalytic activity. We show how volcano plots can be utilized for the rapid screening of prospective Ae catalysts to establish a guideline to achieve maximum activity in facilitating the hydrogenation process.

**Supplementary Information:**

The online version of this article at 10.1007/s11244-021-01480-7.

## Introduction

Hydrogenation of unsaturated bonds is one of the most fundamental transformations in chemistry finding broad applications at every scale of chemical production [1]. With the conventional hydrogenation catalysts utilizing rare, expensive, and often toxic transition metals, there is a great incentive for chemists to find cheaper and environmentally friendly alternatives. In this context, the recent development of the alkaline earth (Ae) metal amides as effective hydrogenation catalysts for various alkenes and imines is especially significant [2–6]. In fact, these catalysts facilitate direct hydrogenation of alkenes under mild conditions while successfully tackling crucial issues pertinent to hydrogenation protocols such as suppressing polymerization and tolerating various functional groups. Strikingly, Ae metal complexes also catalyze highly selective alkene transfer hydrogenation, using 1,4-cyclohexadiene as a reducing agent [7]. Transfer hydrogenation using alternative hydrogen sources is very attractive since it does not require hazardous pressurized H$_2$ or elaborate experimental set-ups [8]. Very recently, efficient hydrogenation with a wide range of substrates was also achieved with ligand-free metallic barium [9]. These ground-breaking results confirm the versatility of simple Ae metal complexes in catalysis, establishing a sustainable alternative to transition metal catalysis for industrial applications.

**Scheme 1:**
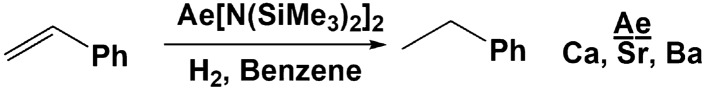
Alkaline-earth metal-catalyzed hydrogenation of styrene to ethylbenzene

Initially, the catalytic activity of the Ae[(NSiMe$_3$)]$_2$ catalysts were demonstrated with their ability to hydrogenate a broad range of alkenes and imines [2–4, 7]. While Mg is found to be inactive for hydrogenation of alkenes, heavier Ae-amides exhibit high catalytic activity. Interestingly, alkali (Ak) amides are also capable of facilitating this transformation although their activity is much lower than the Ae-amides. Combined experimental/computational studies by Bauer et al. firmly established the mechanism of such Ae-catalysed hydrogenation processes proceeding through a metal hydride mediated route.[2, 3] Interestingly, further improvements on activity and substrate scope were brought upon by introducing bulkier amide ligands, e.g. N(TRIP)$_2$ and N(TRIP)(DIPP) (where TRIP=Si*i*Pr$_3$ and DIPP=2,6-(*i*Pr)$_2$C$_6$H$_3$) instead of N(SiMe$_3$)$_2$ [4]. Such observations are rationalized in terms of increased concentration of *in situ* generated catalytically active smaller metal hydride species due to large ligand size.

**Fig. 1:**
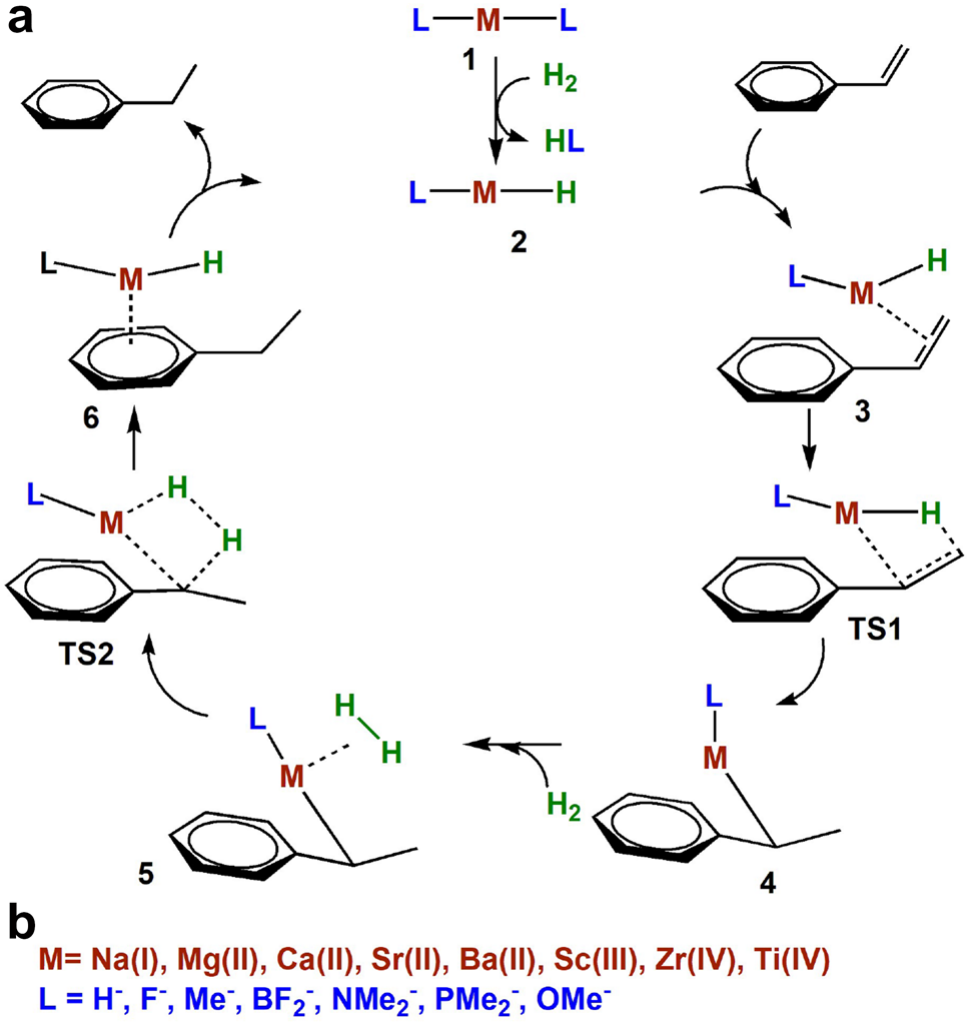
**a** Proposed catalytic cycle for the hydrogenation of styrene to ethylbenzene. **b** The metals and ligands chosen to construct the scaling relationships in this work

Despite the well-established mechanistic picture, a clear-cut understanding of how different Ae metal-ligand combinations influence the catalytic activity is currently lacking. Such knowledge is crucial for the systematic development of Ae-hydrogenation catalysis. One particular tool that can particularly aid in such an understanding of the overall trends in catalytic behavior is molecular volcano plots. Over the past few years, our research group has been developing and utilizing these plots to screen homogeneous catalysts for several important chemical processes [10–18]. Molecular volcano plots predict the performance of a catalyst (in terms of turn over frequencies or a particular energetic criterion) based on an easily computed descriptor variable. These plots are constructed after post-processing the linear free energy scaling relationships (LFESRs) [19–21] obtained between the descriptor and the relative stability of the reaction intermediates and transition states. The most promising catalysts are then easily identified by visually inspecting their location in the plot (appearing near the volcano top or near the plateau). Inspired by this recent development in Ae metal catalysis, here we investigate main group metal-catalyzed hydrogenation of alkenes using molecular volcano plots to uncover the influence of the metal/ligands and their interplay on the energetics of the catalytic cycle.

## Results and Discussion

**Fig. 2:**
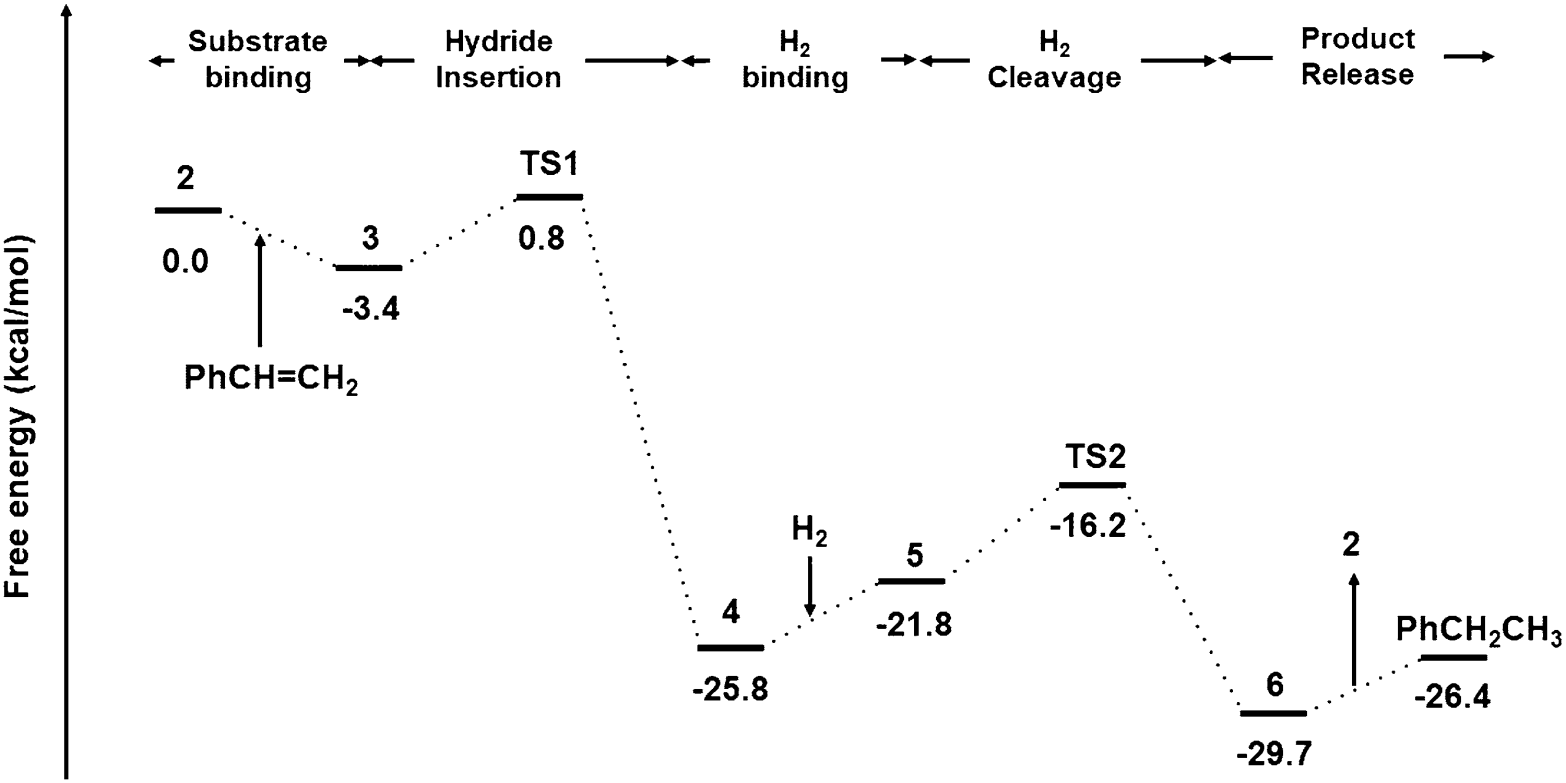
Overview of the catalytic cycle for the hydrogenation of styrene to ethylbenzene by Ca(NMe$_2$)$_2$

*Mechanism* The first step to construct a molecular volcano plot is to settle the mechanism of the corresponding catalytic cycle (Scheme 1). Bauer et al. already investigated the mechanism of direct hydrogenation of styrene by Ca(N(SiMe$_3$))$_2$ through DFT computations (Fig. 1) [2]. First, the precatalyst **1** exchanges an amide ligand for a hydride ligand to yield **2**, which is the entry point into the catalytic cycle. Hydrogenation begins with the coordination of the alkene substrate to the catalyst leading to the formation of **3**. The Ae-H bond then inserts into styrene to produce the benzylmetal intermediate **4** via **TS1**. The precomplex, **5**, precedes the heterolytic cleavage of H$_2$ through **TS2** leading to **6** which upon dissociation releases ethylbenzene and regenerates the catalyst, **2**. Figure 2 illustrates a representative free energy profile for the catalytic hydrogenation process (along with the key intermediates and transition states) using a model catalyst, Ca(NMe$_2$)$_2$.

*LFESRs* The prerequisite to constructing a volcano plot is to establish LFESRs which ascertain that the free energies of the various catalytic cycle intermediates are correlated. The LFESRs were determined by analyzing a set of 54 catalysts produced from the combinations of 8 metal cations (Na$^+$, Mg$^{2+}$, Ca$^{2+}$, Sr$^{2+}$, Ba$^{2+}$, Sc$^{3+}$, Ti$^{4+}$, Zr$^{4+}$) and 7 small anionic ligands of varying connecting atom identities (H$^-$, F$^-$, BF$_2$ $^-$, Me$^-$, NMe$_2$ $^-$, PMe$_2$ $^-$, OMe$^-$) [22]. Apart from the mono/bivalent metals, tri and tetravalent metals are chosen additionally as a part of a systematic investigation of the catalytic cycle to reveal general trends in reactivity [23].

To ensure general trends in the scaling relationships, the entry point to the catalytic cycle is considered to be a monomeric bisligated species. Therefore, the effects of ligand over-coordination, catalyst aggregations, and the possible deactivation channels are not considered here.

**Scheme 2:**
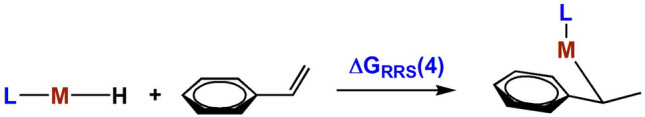
Estimation of the descriptor variable ($\Delta$G$_{RRS}$(**4**)) as the relative stability of intermediate **4**

**Fig. 3:**
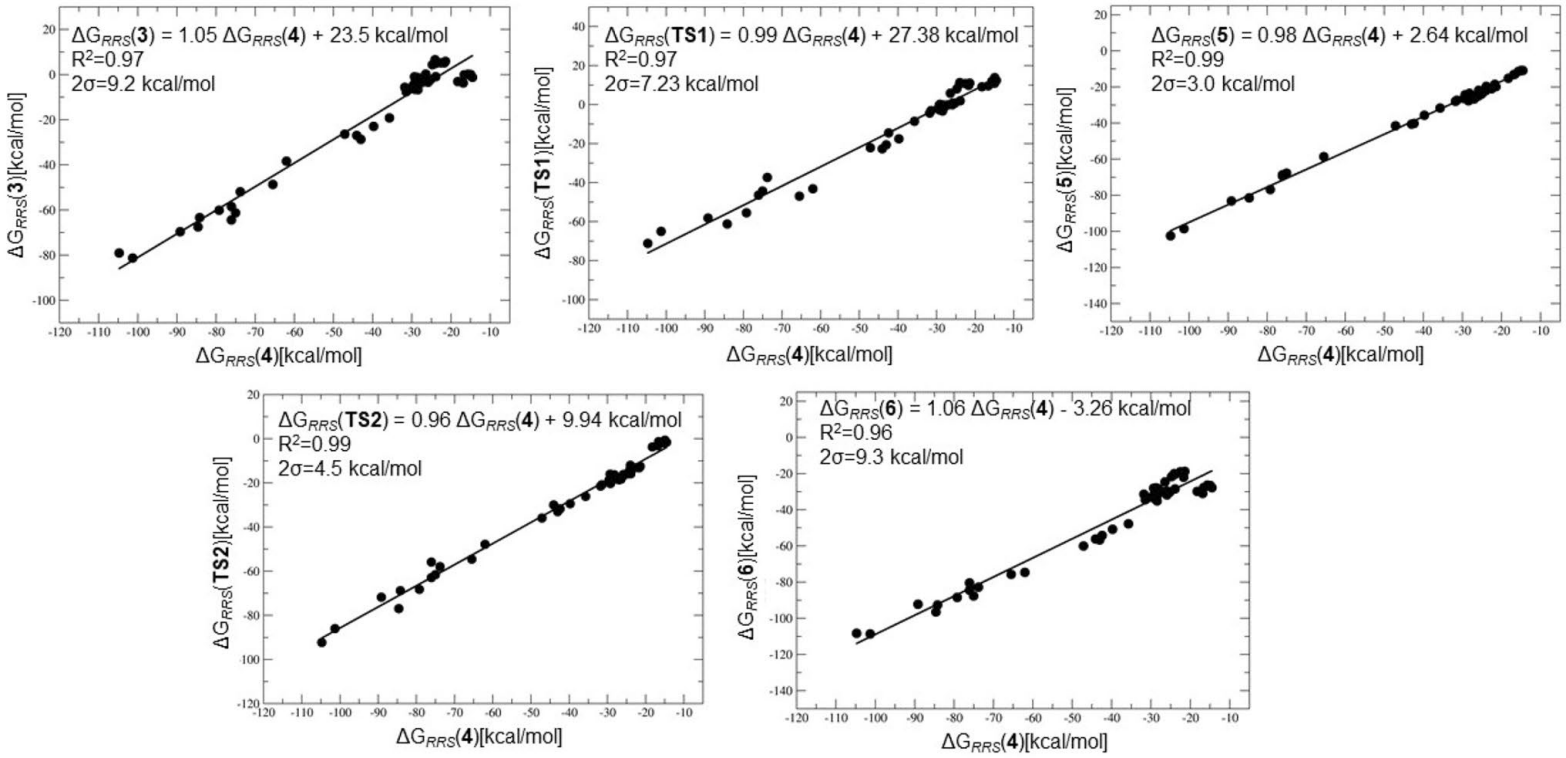
Linear free energy scaling relationships between the descriptor variable $\Delta$G$_{RRS}$(**4**) and the catalytic cycle intermediates and transition states

We computed the full catalytic cycle for all 54 catalysts using standard DFT computations (see Computational Details) and the free energies associated with the intermediates and transition states (I/TSs) are estimated **r**elative to the **r**eference **s**tate ($\Delta$G$_{RRS}$), **2**, which is the entry point to the catalytic cycle. Based on the quality of the linear correlations, $\Delta$G$_{RRS}$(**4**) is chosen as the descriptor variable for the volcano analyses (see Scheme 2). The computed LFESRs along with the quality of the linear fits, established with $\Delta$G$_{RRS}$(**4**) as a descriptor, are depicted in Fig. 3.

**Fig. 4:**
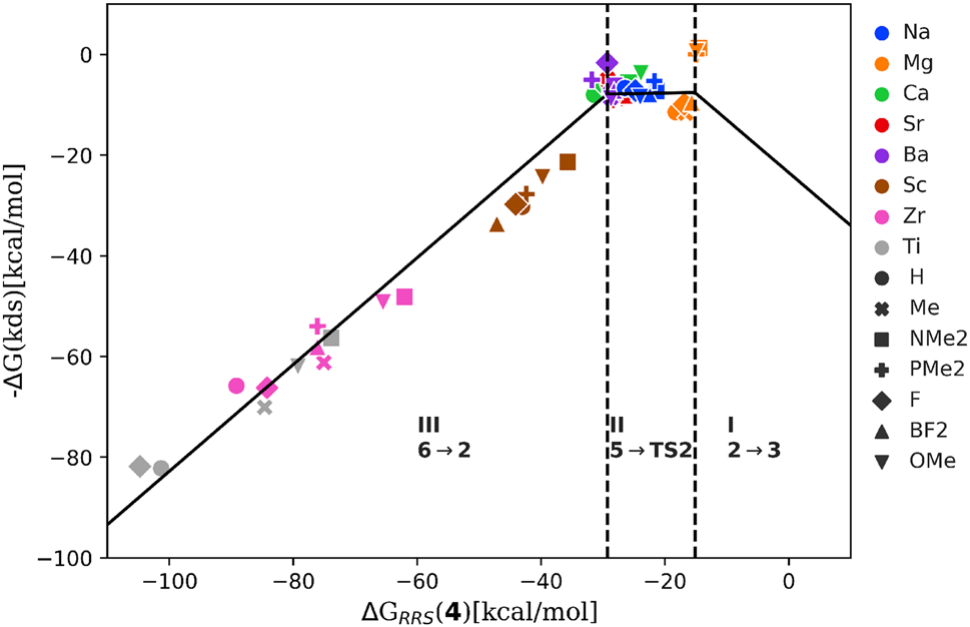
Volcano plot that demonstrates the expected activity of the catalysts by predicting the value of the most energetically costly reaction step in the hydrogenation of styrene to ethylbenzene. The dashed lines indicate a change in the most difficult reaction step in the mechanism

*Kinetic volcano plot* The LFESRs are post-processed to obtain the kinetic volcano plot [10, 11] in Fig. 4. The descriptor $\Delta$G$_{RRS}$(**4**) is plotted along the x-axis and the y-axis corresponds to the free energy required to complete the most difficult reaction step, kinetics determining step (kds). The plot can be divided into three areas, which correspond to different limiting reaction steps. The limiting reaction in region-I is the binding of styrene (**2**$\rightarrow$**3**), while the region-III is limited by the release of ethylbenzene (**6**$\rightarrow$**2**) from the catalyst. In line with Sabatier’s principle terminology, regions-I and III can be readily interpreted as the weak and strong binding sides that characterize the right and left slopes of the volcano, respectively. Region-II (the plateau of the volcano), on the other hand, corresponds to the most kinetically balanced situation in which the most energetic step involves the barrier associated with the heterolytic H$_2$ cleavage(**5**$\rightarrow$**TS2**). Catalysts occupying this region are especially interesting as they nearly fulfill Sabatier’s definition of an ideal catalyst.

**Fig. 5:**
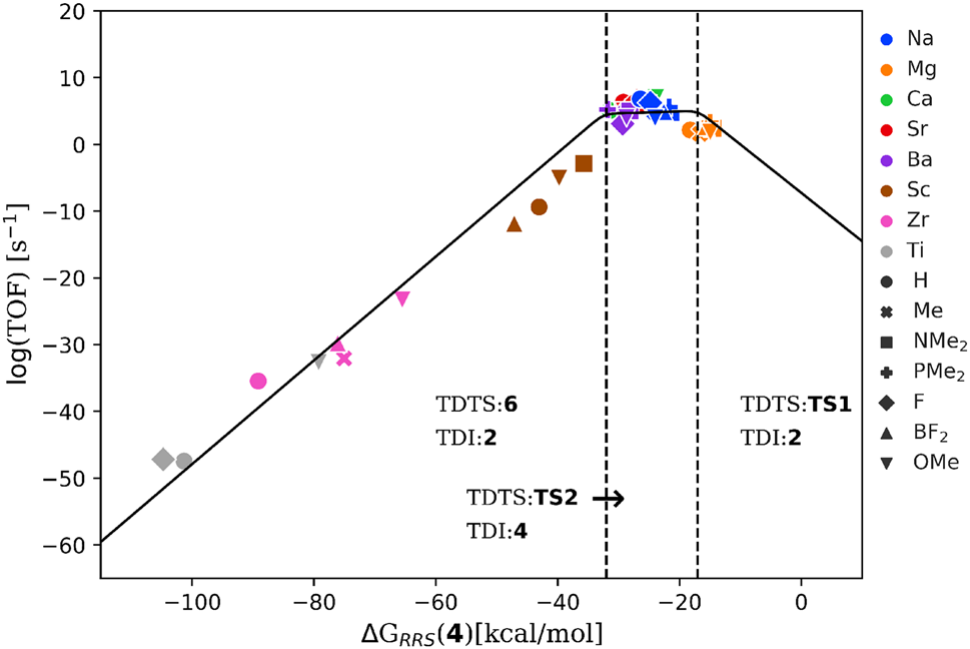
TOF volcano plot for hydrogenation of styrene to ethylbenzene. The dashed lines represent changes in the nature of the TDI and TDTS in the catalytic cycle. (TOF: Turnover Frequency; TDI: Turnover Determining Intermediate; TDTS: Turnover Determining TS)

*TOF volcano plot* Instead of kds, which involves reaction energies between consecutive two intermediates/TSs, the catalytic activity could be expressed in terms of more realistic turnover frequencies (TOFs) [24] by examining the free energy profiles of the catalysts under the framework of Eyring’s transition state theory (TST). We utilized LFESRs in Fig. 5 to determine hypothetical free energy profiles for a range of descriptor values ($-115$ to 10 kcal/mol) and these were used as input to derive theoretical TOF values corresponding to each descriptor value. When plotted with a logarithmic y-axis, the TOF volcano takes a more or less similar shape as that of the kinetic volcano (see Fig. 4). The TOF volcano in Fig. 5 is best interpreted using the energy span model [25] (microkinetic analysis) which associates different regions of the plot with a certain pair of intermediate (TDI: turnover determining intermediate) and transition states (TDTS: turnover determining TS), or, two intermediates for a thermodynamically driven association or dissociation process leading to the largest barrier in a free energy profile. The overall energy span associated with a catalytic cycle is typically defined by the following equations,

1 $$\begin{aligned}&\delta E= max_{i,j}(T_i - I_j + \delta G_{ij} )\end{aligned}$$

2 $$\begin{aligned}&\delta G_{ij}= 0\,(\,for\,T_i\,after\, I_j)\;or,\nonumber \\&\quad \Delta G_R\,(\,for\,T_i\,before\, I_j) \end{aligned}$$

where $T_i$ and $I_j$ are the Gibbs free energies of the *i*th TS and *j*th intermediates in the profile, respectively, and $\Delta G_R$ is the Gibbs free energy of the reaction. Importantly, the TDI and TDTS are not necessarily the highest and lowest states, nor do they have to be adjoined as a single step. The energy difference between the TDI and the TDTS is the apparent activation energy of the entire catalytic cycle determining the catalytic efficiency.

Applying the energy span model, the TOF volcano plot can be divided into three regions depending on the change in TDI/TDTS as a function of the descriptor. The right side region (equivalent to region-I in Fig. 4) corresponds to **TS1** as TDTS and **2** as TDI for descriptor values $\Delta$G$_{RRS}$(**4**) $> -16$ kcal/mol. For the top region (equivalent to region-II in Fig. 4) with descriptor values lying between $-16$ to $-29$ kcal/mol, TDI and TDTS change to **4** and **TS2**, respectively. Finally, for $\Delta$G$_{RRS}$(**4**) $< -29$ kcal/mol (equivalent to region-III in Fig. 4) the TDTS and TDI become **2** and **6** [26]. Thus, for the candidates appearing on the right region, the catalysts remain strongly bound to the substrate resulting in a high energy penalty for the hydride insertion step. On the other hand, the catalysts falling on the left slope of the volcano have a overly stabilized intermediate, **6** which makes the product release step strongly endergonic. Approaching the top of the volcano from either side corresponds to balancing the energy requirements of these two steps and increasing the catalytic activity.

*Understanding the Influence of Metal and Ligands* To examine the potential of the catalysts for the hydrogenation of styrene, we plotted each catalyst candidate according to their descriptor variable ($\Delta$G$_{RRS}$(**4**)) on the kinetic as well as on the TOF volcano. The position of a catalyst determines its limiting reaction steps (Fig. 4) or a pair of TDTS and TDI (Fig. 5) within an energy span framework. Nearly all the catalysts considered in the work appear either in region-II or III (TDTS/TDI: **6**/**2** or **TS2**/**4**) of the kinetic (TOF) volcano plot. Their relative positioning suggests that they are roughly clustered based on the metal oxidation state and the total charge of the catalyst. Ae/Ak catalysts with a formally bi/mono positive medium to large size metal ions (Na$^+$, Ca$^{2+}$, Sr$^{2+}$, and Ba$^{2+}$) appear at the top of the volcano (*i.e.*, region-II) indicative of their higher activity for styrene hydrogenation. They have nearly ideal kinetic profiles and are limited by the molecular hydrogen cleavage step. Note that the experimentally inactive Mg catalysts separate from the clusters of the other Ae candidates and lie closer to the intersecting area I and II. A careful examination of the structure of the intermediate **4** reveals that, unlike other Ae cations, Mg$^{2+}$ binds to the benzyl anion in more of a monodentate fashion (involving the benzylic carbon) presumably due to the smaller size of the Mg$^{2+}$ cation. In fact, the other bigger metal cations (Ca, Sr and Ba) in the Ae catalysts family for which the benzyl anion offers a bidentate binding mode (involving both the *ipso* and the benzylic carbon centers) making the corresponding benzylmetal complexes more stabilized compared to their Mg-counterpart. As shown in Fig. 5, Mg candidates are anticipated to be somewhat limited by the formation of **TS1** (TDTS/TDI: **TS1**/**2**), the hydride insertion step, but still predicted as fairly active (although less active compared to other Ae catalysts ). Overall, these findings imply a slightly different behavior of Mg compared to other Ae metals.[27]

**Fig. 6:**
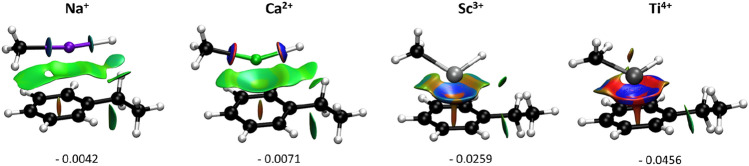
NCI analysis of intermediate **6** for catalysts featuring mono-, bi-, tri- and tetravalent cations combined to $\hbox {Me}^{-}$. The gradient isosurfaces (s = 0.05 a.u.) are colored on a BGR scale according to sign$(\lambda _2)\rho$ over the range $-0.03$ to 0.03 a.u. The values of the attractive peak denoting the interaction between the cation and the product are also displayed. Red isosurfaces stand for repulsive interactions while on the other hand blue isosurfaces indicate attractive interactions. Green indicates van der Waals-type interactions

Highly charged cations (tri- or tetravalent Sc, Ti and Zr) lead to a more strongly stabilized (more negative $\Delta$G$_{RRS}$(**4**)) benzylmetal intermediate than the Ae cations. Accordingly, all tri- or tetravalent Sc, Ti, and Zr-containing candidates fall on the region-III being limited by the release of the product. All of these catalysts overstabilize complex **6** and thus show minimal catalytic activity. A crucial structural feature of **6** is the cation-$\pi$ interaction in which the central metal cation is bound to the phenyl ring of ethylbenzene. Further insights into this interaction can be obtained by estimating the noncovalent interaction (NCI) index of the intermediate **6** (see SI for computational details of NCI analysis). Figure 6 illustrates the NCI isosurfaces computed for a few representative metal-ligand combinations. All complexes exhibit evidence for attractive interaction between the cation and the product. For a given ligand (Me$^-$), the cation-$\pi$ interaction strengthens with an increased charge of the central metal cation, and thus, highly charged cations lead to the strongest interactions rendering the release of the product more difficult. The corresponding interactions for bivalent metals are nearly similar (see Fig. S6 and S7). In fact, these metals delicately regulate the cation-$\pi$ interaction so that the energetic cost of association of styrene and dissociation of ethylbenzene from the catalyst balance each other, which is key to achieve high TOF values. The reason for the different behavior of Mg might be related to its small size preventing multiple non-bonding contacts essential for a strong cation-$\pi$ interaction. Overall, the relative activity of the catalyst candidates increases in the order of tetravalent < trivalent < bi or, monovalent metals. Experimentally, there are substantial activity differences among Ca, Sr and Ba catalysts although based on the catalytic cycle in Scheme 1b, these three metals are predicted to exhibit comparable reactivity. These results likely indicate that the observed reactivity trend in experiment stems from several side reactions pertinent to the catalytic cycle.

In contrast to the metals, the effect of the ligands on the catalytic activity is much less significant. The electron-withdrawing (EW) ligands tend to strengthen the metal-benzyl interaction hence stabilizing **4** (more negative value of the descriptor). For instance, F/BF$_2$ stabilizes **4** more than electron-donating (ED) ligands such as NMe$_2$/OMe. Thus, given a particular metal ion, EW ligand bearing candidates progressively appear towards the left-hand side of the volcano. While the manifestation of this effect is only minor for the bi/monovalent cations, this is amplified when the ligands are combined with highly positive cations (Ti$^{4+}$ and Zr$^{4+}$). The latter exhibits fairly different activity, while replacing F with NMe$_2$ producing a substantial shift toward the weak binding side (*i.e.* right along the x-axis). As shown in Figs. S4 and S5, the corresponding NCI analyses are consistent with these results. These observations suggest that it might not be an effective strategy to improve the intrinsic activity of the Ae catalysts through ligand tuning, often a preferred option in TM-based catalysis. Nevertheless, it should be noted that the choice of ligands could have an impact on the overall performance of the catalytic cycle by influencing the side reactions or catalyst-aggregation [4]. The metal charge and size are the most relevant factors affecting the Ae catalysts’ activity towards hydrogenation.

## Conclusions

In summary, the performance of main group metal catalysts for the hydrogenation of styrene was examined using molecular volcano plots. Our findings reveal that the activity of the metal is essentially determined by the formal charge and size of the metal cation. Catalysts with highly charged cations engage in stronger interactions with styrene mainly through cation-$\pi$ interactions that limits the release of the product. The hard Mg$^{2+}$ cation tends to disfavor the hydride insertion. Finally, medium to large mono- and bivalent metals provide the ideal balance for alkene hydrogenation. The influence of the ligands is important when they are combined with tri/tetravalent metals. The intrinsic activity of the Ae catalysts is not significantly affected by the choice of the ligands and hence, most of these are predicted to have comparable performance towards styrene hydrogenation.

## Computational Details

The geometries of all catalytic cycle intermediates and TSs were optimized at the M06 [28, 29]/def2-SVPD [30] level in implicit benzene solvent using Gaussian16, A.03 [31]. The M06 hybrid functional was proven to be quite accurate for main group thermochemistry and kinetics and our previous works demonstrated that this level of theory is adequate to support the experimental findings of imine and alkene hydrogenations by Ae amides.[2, 3] An analysis of the harmonic vibrational frequencies was performed to ascertain the nature of each optimized structures either as a minimum (no imaginary frequency) or a transition state (one imaginary frequency). Single point energies were computed on the M06 geometries at the PBE0-dDsC/TZ2P [32–37] level as implemented in ADF [38]. Free energy corrections were obtained at the M06/def2-SVPD level using the rigid-rotor harmonic oscillator model within the Goodvibes [39] program developed by Paton and Funes-Ardoiz. Solvation corrections for the reported free energy values were obtained using COSMO-RS solvation model at PBE0-dDsC/TZ2P level in benzene [40]. The turnover frequencies (TOF) were calculated at 298.15 K and a concentration of 1 M using AUTOF program developed by Uhe, Kozuch, and Shaik [41–43]. The NCI plots were computed with the NCIPLOT program, starting from the M06 wave functions of the optimized geometries [44]. Additional computational details for NCI analysis are provided in the SI.

## Supplementary Information

Below is the link to the electronic supplementary material.

Electronic supplementary material 1 (PDF 2179 kb)

Electronic supplementary material 2 (XLSX 90 kb)

Electronic supplementary material 3 (XYZ 380 kb)

## Declarations

### Supporting Information

Additional computational details and figures, NCI analysis, and free energy profiles for all catalysts (PDF) Computed electronic energies, free energy corrections, solvent corrections, and total free energies (XLS) Cartesian coordinates of all computed structures in XYZ format (ZIP)
