# Supplementary material for: Uncovering the Activity of Alkaline Earth Metal Hydrogenation Catalysis Through Molecular Volcano Plots
Source: Top Catal. 2021 Aug 25;65(1-4):289–95. doi: 10.1007/s11244-021-01480-7 (PMC8816741; doi:10.1007/s11244-021-01480-7)
Supplement: Supplementary file 1 — Electronic supplementary material 1 (PDF 2179 kb) [file 11244_2021_1480_MOESM1_ESM.pdf]

Supporting Information for Uncovering the Activity of Alkaline Earth Metal Hydrogenation Catalysis through Molecular Volcano Plots

Shubhajit Das,† Bart De Tobel,‡ Mercedes Alonso,‡ and Clémence Corminboeuf*,†

†*Laboratory for Computational Molecular Design, Institute of Chemical Sciences and Engineering, Ecole Polytechnique Fedéralé de Lausanne (EPFL), 1015 Lausanne, Switzerland*

‡*Eenheid Algemene Chemie (ALGC), Vrije Universiteit Brussel (VUB), Pleinlaan 2, 1050 Brussels, Belgium*

E-mail: clemence.corminboeuf@epfl.ch

Construction of Molecular Volcano Plots

A comprehensive description of the different steps involved in the building of molecular volcano plots are discussed elsewhere. For the detailed methods of construction of the kinetic and TOF volcano plots, interested readers are referred to the following two references.

Kinetic: Wodrich, M. D.; Busch, M.; Corminboeuf, C. Accessing and predicting the kinetic profiles of homogeneous catalysts from volcano plots. Chemical science **2016**, 7, 5723–5735.

TOF: Wodrich, M. D.; Sawatlon, B.; Solel, E.; Kozuch, S.; Corminboeuf, C. Activity-based screening of homogeneous catalysts through the rapid assessment of theoretically derived turnover frequencies. ACS Catalysis **2019**, 9, 5716–5725.

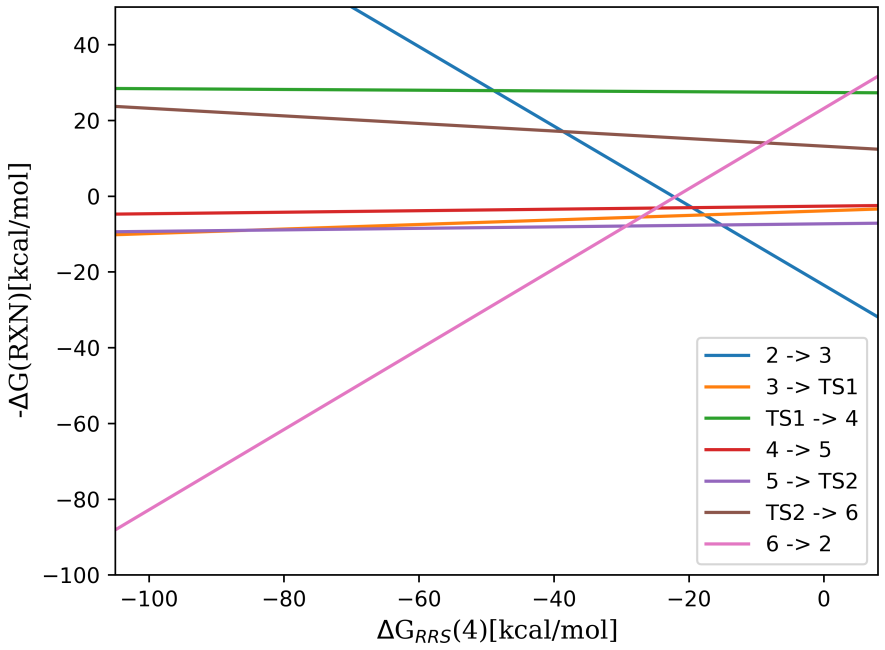

Figure S1: Stoichiometric reaction profile for main group metal-catalyzed hydrogenation of styrene to ethylbenzene

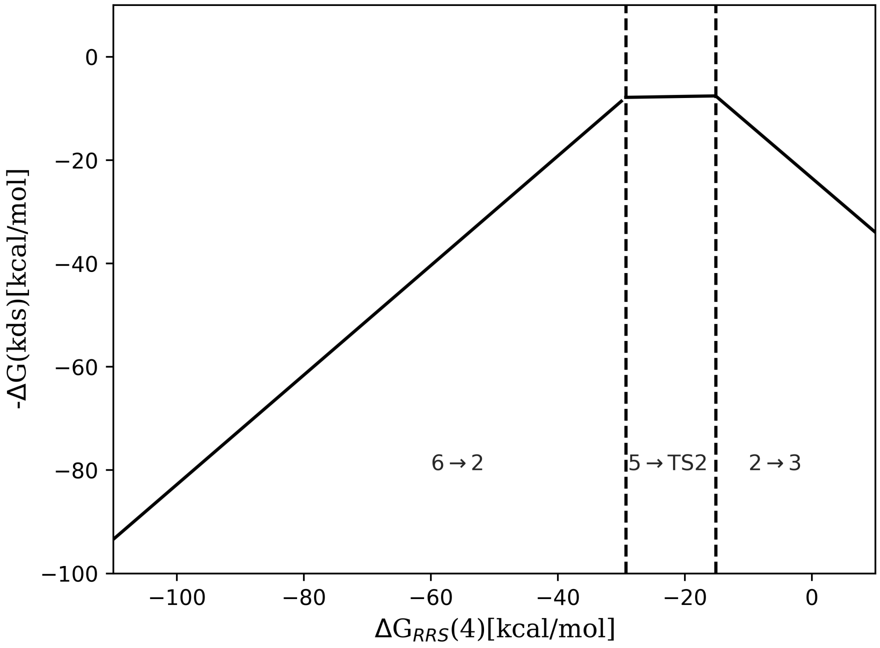

Figure S2: Kinetic volcano plot for main-group metal catalyzed hydrogenation of styrene

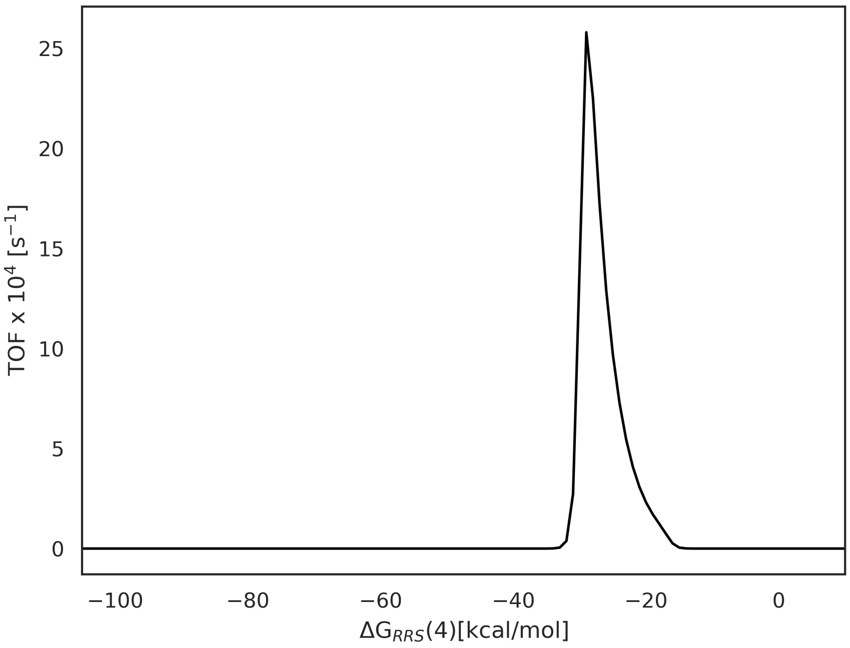

Figure S3: TOF volcano plot (linear axis) for hydrogenation of styrene to ethylbenzene.

Additional computational details

The NCI plots were computed with the NCIPLOT program, starting from the M06 wave functions of the optimized geometries.[1] The NCI index is based on the electron density and its derivatives. The relationship between the reduced density (s) and the electron density (ρ) is:

$$s = \frac{1}{2(3\pi^2)^{1/3}} \frac{|\nabla \rho|}{\rho^{4/3}} \tag{1}$$

When representing s versus ρ, the presence of non-covalent interactions is shown by the characteristic peaks at low density values, originating from the annihilation of the density gradient at these points.[2] The reduced density gradient and the electron density are evaluated at a set of grid points around the system of interest. The sign of the second eigenvalue (λ_2) of the electron-density Hessian matrix allows to distinguish between the type of interaction. A negative eigenvalue ($\lambda_2 < 0$) denotes a bonding interaction, while a positive eigenvalue ($\lambda_2 > 0$) characterizes nonbonding interactions. The strength of the interaction is derived from the density values of the low-gradient spikes. The Laplacian sign is a widely used tool to distinguish between different types of strong interactions.[3]Bonded interactions give rise to a charge accumulation ($\nabla^2 \rho < 0$), whereas a density depletion is the result from antibonding interactions ($\nabla^2 \rho > 0$). For weaker, noncovalent interactions, the Laplacian in the interatomic region is dominated by the positive contribution for both bonding and nonbonding interactions. Therefore, to distinguish between attractive and repulsive interactions, one must consider density accumulation or depletion in the plane perpendicular to the interaction. This is mainly characterized by the second eigenvalue (λ_2) of the electron-density matrix. The Laplacian can be written as follows, using the three components of the maximal variation.

$$\nabla^2 \rho = \lambda_1 + \lambda_2 + \lambda_3 \tag{2}$$

In this equation, the λ_i values are the three eigenvalues of the electron-density Hessian matrix. Since it is the sign of the second eigenvalue that is indicative for the type of

interaction, the density gradient is plotted against the product of the sign(λ_2) and the electron density ρ. The visualization of the gradient isosurface in real space is a useful tool for visualizing non-covalent interactions. The value of the sign(λ_2)ρ is used to colour the different isosurfaces. In general, a RGB (red-green-blue) scale is used. Red isosurfaces stand for repulsive interactions while on the other hand blue isosurfaces indicate attractive interactions. Green indicates van der Waals-type interactions.

NCI analysis of intermediate 6

To obtain further insight into the cation-π interactions (CPI), the NCI index has been computed for different metal complexes of intermediate **6**. To evaluate the influence of the metal, the ligand (Me^-) was kept constant. For all complexes, a nonbonding interaction is located at the ring center of the substrate (see Figure 6, S4, and S6). Additionally, an attractive and repulsive spike in the s(ρ) diagram was localized for the cation-π interaction (CPI) between the metal and the product. For the same substrate, the CPI weakens significantly with a decreasing cation charge. The shift in the peak is largest between the tetra-, and trivalent, and the tri- and divalent cations. This validates the hypothesis that highly charged cations are limited by product release, due to the strong CPI with the Ph ring of ethylbenzene, leading to an overstabilization of complex **6**. The shift in the peak describing the metal-product interaction is rather small when going from Ca^{2+} to Na^+, while a large shift (Figure S5) is visible in the peak that is responsible for the interaction between the cation and the Me^- ligand and the cation and the hydride. The positions of the low gradient spikes corresponding to the CPI for evenly charged cations are very similar. Figure S6 and S7 provide the NCI analysis for a few representative catalysts containing Ae metals.

To evaluate the influence of the ligand on the substrate activity, we looked at the NCI analysis of two Ti^{4+} complexes, since the ligand effect was expected to be amplified for highly charged cations. A small shift towards higher density values (Figure S4 and S5) was

observed for the attractive peak of the CPI when changing from a more electron-donating ligand like NMe_2^- towards an electron-withdrawing ligand like F^-. As expected, the NCI analysis validates that the metal charge plays a more crucial role in determining the activity of the catalysts towards the hydrogenation.

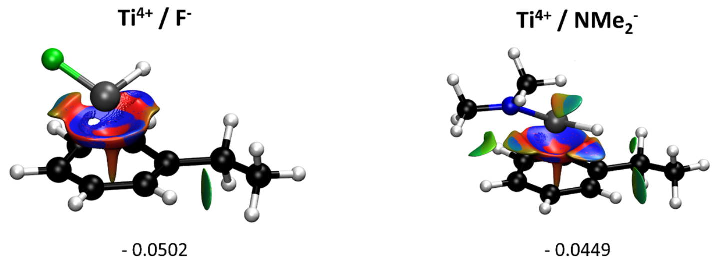

Figure S4: NCI analysis of intermediate **6** for two representative Ti^{4+} / L^- combinations. The gradient isosurfaces (s =0.05 a.u.) are coloured on a BGR scale according to sign$(\lambda_2)\rho$ over the range -0.03 to 0.03 a.u. The ρ values of the attractive peak denoting the interaction between the cation and the product are also displayed.

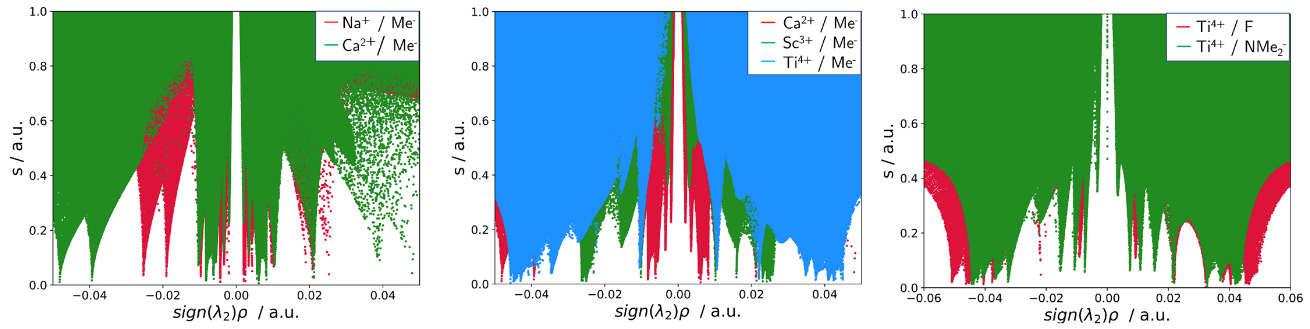

Figure S5: NCI analysis of intermediate **6** for different metal-ligand combinations.

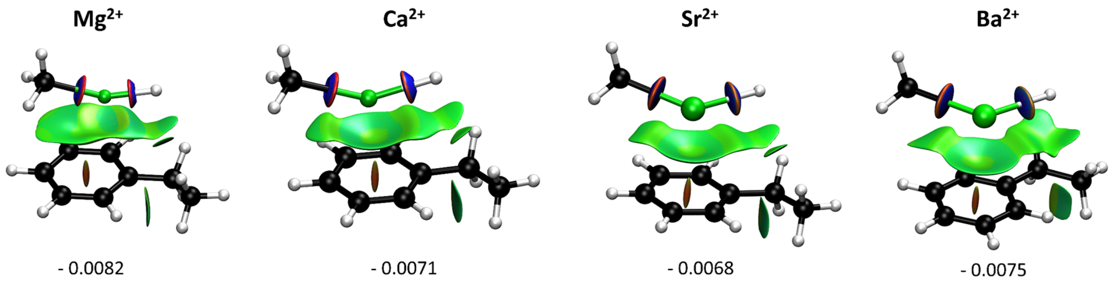

Figure S6: NCI analysis of intermediate **6** for different Ae metals combined with Me^-. The gradient isosurfaces (s =0.05 a.u.) are coloured on a BGR scale according to sign$(\lambda_2)\rho$ over the range -0.03 to 0.03 a.u. The ρ values of the attractive peak denoting the interaction between the cation and the substrate are also displayed.

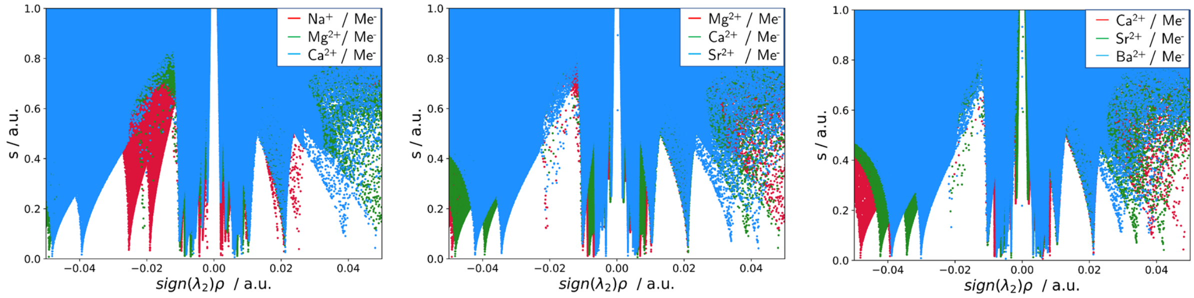

Figure S7: NCI analysis of intermediate **6** for different mono-, bivalent metals combined with Me^-.

References

(1) Contreras-García, J.; Johnson, E. R.; Keinan, S.; Chaudret, R.; Piquemal, J.-P.; Beratan, D. N.; Yang, W. NCIPLOT: a program for plotting noncovalent interaction regions. *Journal of Chemical Theory and Computation* **2011**, *7*, 625–632.

(2) Johnson, E. R.; Keinan, S.; Mori-Sánchez, P.; Contreras-García, J.; Cohen, A. J.; Yang, W. Revealing noncovalent interactions. *Journal of the American Chemical Society* **2010**, *132*, 6498–6506.

(3) Bader, R. F.; Essén, H. The characterization of atomic interactions. *The Journal of Chemical Physics* **1984**, *80*, 1943–1960.

Tables S1: Free energy profiles of the catalytic hydrogenation cycles for all catalysts

**M**	**L**	**3**	**TS1**	**4**	**5**	**TS2**	**6**
Mg	H	−3.10	9.11	−18.23	−15.22	−3.73	−29.83
Mg	Me	−0.08	9.61	−16.62	−13.03	−1.30	−27.72
Mg	NMe_2	−1.26	12.34	−14.51	−10.85	−1.62	−27.82
Mg	PMe_2	0.03	10.89	−14.97	−11.06	−1.97	−27.11
Mg	F	−3.70		−16.87	−13.18	−3.28	−30.97
Mg	BF_2	0.14	11.92	−15.66	−11.42	−1.84	−26.41
Mg	OMe	−0.61	13.83	−14.93	−10.78	−0.67	−26.58
Ca	H	−7.67	−3.14	−31.43	−27.36	−20.87	−34.46
Ca	Me	−3.25	−0.55	−28.74	−25.00	−18.10	−32.16
Ca	NMe_2	−3.43	0.83	−25.85	−21.86	−16.23	−29.70
Ca	PMe_2	−5.34	−1.37	−28.40		−17.05	−32.15
Ca	F	−4.67	−2.07	−29.63	−26.42	−18.89	−32.76
Ca	BF_2	−4.19	−1.07	−28.33	−25.26	−17.55	−30.77
Ca	OMe	−0.93	1.91	−23.87	−19.67	−15.91	−28.50
Sr	H	−6.54	−2.77	−29.21	−25.09	−20.35	−33.63
Sr	Me	−3.72	−0.50	−28.71	−25.37	−17.83	−29.85
Sr	NMe_2	−2.21	0.78	−25.20	−24.15	−16.01	−30.23
Sr	PMe_2	−6.67	−3.32	−28.45	−27.64	−18.55	−35.16
Sr	F	−3.27	−0.16	−26.98	−26.69	−18.66	−30.06
Sr	BF_2	−2.48	−0.19	−25.92	−25.01	−16.85	−31.80
Sr	OMe	−3.27	0.08	−29.10	−26.29	−18.44	−30.46
Ba	H	−2.88	−2.89	−28.78	−25.56	−18.10	−28.76
Ba	Me	−2.81	−2.35	−28.74	−24.94	−16.32	−29.75
Ba	NMe_2	−1.54	−0.25	−28.16	−23.69	−17.48	−28.36
Ba	PMe_2	−5.65	−4.35	−31.80	−27.97	−21.45	−31.49
Ba	F	−0.98	−0.28	−29.32	−24.36	−16.06	−28.12
Ba	BF_2		−1.95	−28.21	−23.52	−16.32	−28.31
Ba	OMe	−1.25	−0.22	−28.70	−25.50	−16.65	−27.93
Sc	H	−28.75	−20.62	−43.04	−40.57	−33.05	−56.71
Sc	Me	−24.39	−18.10				−53.37
Sc	NMe_2	−19.20	−8.63	−35.70	−31.69	−26.14	−47.82
Sc	PMe_2		−14.58	−42.38	−40.20	−31.74	−54.22
Sc	F	−26.98	−22.69	−44.07		−29.98	−56.22
Sc	BF_2	−26.40	−22.17	−47.11	−41.52	−35.96	−60.06
Sc	OMe	−22.97	−17.57	−39.75	−35.70	−29.46	−50.85
Zr	H	−69.65	−58.16	−89.14	−83.20	−71.82	−92.26
Zr	Me	−61.28	−44.36	−75.04	−67.79	−61.60	−87.65
Zr	NMe_2	−38.40	−43.16	−62.02		−47.84	−74.63
Zr	PMe_2	−64.44		−76.08	−69.37	−62.93	−80.48
Zr	F	−63.39	−61.23	−84.20		−68.84	−92.66
Zr	BF_2	−58.47	−46.40	−76.08	−68.67	−55.89	−84.49
Zr	OMe	−48.68	−46.99	−65.49	−58.62	−54.58	−75.72
Ti	H	−81.32	−65.03	−101.31	−98.58	−86.02	−108.65
Ti	Me	−67.49		−84.59	−81.47	−76.98	−96.54
Ti	NMe_2	−51.97	−37.37	−73.83		−57.99	−82.78
Ti	F	−79.05	−71.22	−104.73	−102.44	−92.36	−108.27
Ti	OMe	−60.11	−55.48	−79.22	−76.85	−68.24	−88.48
Na	H	0.23	5.78	−26.43	−25.04	−18.32	−24.64
Na	Me	6.57	10.50	−24.07	−22.05	−13.75	−20.01
Na	NMe_2	5.81	10.97	−21.43	−19.80	−12.51	−18.84
Na	PMe_2	5.18	9.81	−21.69	−18.53	−13.18	−22.06
Na	F	4.31	8.01	−24.76	−23.17	−15.96	−21.69
Na	BF_2	5.15	10.73	−22.54	−21.02	−13.13	−19.06
Na	OMe	4.88	11.26	−23.98	−20.57	−12.05	−20.53
